# Prevalence and Intensity of *Cardicola* spp. Infection in Ranched Southern Bluefin Tuna and a Comparison of Diagnostic Methods

**DOI:** 10.3390/pathogens10101248

**Published:** 2021-09-27

**Authors:** Cecilia Power, Shannon Evenden, Kirsten Rough, Claire Webber, Maree Widdicombe, Barbara F. Nowak, Nathan J. Bott

**Affiliations:** 1 School of Science, RMIT University, Bundoora, VIC 3083, Australia; s3328980@student.rmit.edu.au (C.P.); s3599119@student.rmit.edu.au (M.W.); b.nowak@utas.edu.au (B.F.N.); 2 Australian Southern Bluefin Tuna Industry Association, South Quay Blvd, Port Lincoln, SA 5606, Australia; shannon@asbtia.com.au (S.E.); kirstenrough@bigpond.com (K.R.); claire@asbtia.com.au (C.W.)

**Keywords:** *Cardicola*, bluefin tuna, blood fluke, praziquantel, aquaculture, diagnostics

## Abstract

The parasitic blood flukes *Cardicola forsteri* and *C. orientalis* are an ongoing health concern for Southern Bluefin Tuna *Thunnus maccoyii* (SBT) ranched in Australia. In this study we compared the effect of treatment, company, and ranching year on blood fluke infections in ranched SBT. SBT were sampled during the 2018 and 2019 ranching seasons from praziquantel (PZQ) treated pontoons and untreated pontoons managed by two companies. Severity of infection was diagnosed by several criteria including adult fluke counts from hearts, egg counts from gill filaments and the use of specific quantitative polymerase chain reaction (qPCR) for detection of *C. forsteri* and *C. orientalis* ITS-2 DNA in SBT hearts and gills. PZQ treatment remains highly effective against *C. forsteri* infection. Prevalence and intensity of *Cardicola* spp. infection was lower in 2019 than 2018 for Company A in treated pontoons at week 12 and week 17 of ranching, and lower for Company A than Company B in untreated pontoons at month 5 of ranching. Results indicate re-infection may be less likely in the environment near Company A pontoons, and consistent years of treatment may have lowered the parasite load in the environment. qPCR demonstrated higher sensitivity when comparing diagnostic methods for *C. forsteri* in heart, and higher specificity when comparing diagnostic methods for *Cardicola* spp. in gills. Continuing to monitor blood fluke infections in ranched SBT can help to detect changes in drug efficacy over time and help industry to develop a best practice for treatment.

## 1. Introduction

Southern Bluefin Tuna (SBT), *Thunnus maccoyii*, is a commercially important aquaculture species in Australia [1]. Wild juvenile SBT are caught in the Great Australian Bight via purse seine and slowly towed back to the Spencer Gulf aquaculture zone near Port Lincoln, South Australia, where they are transferred to ranching pontoons. Ranched SBT are fattened on baitfish for 2–7 months before harvesting, enabling industry to add quality and value to their catch quotas [2]. An ongoing health concern in ranched SBT is infection by parasitic blood flukes *Cardicola forsteri* and *Cardicola orientalis* (Trematoda: Aporocotylidae) [3,4,5,6].

*Cardicola forsteri* was first described from the hearts of SBT, and *C. orientalis* reported from the branchial arteries of SBT gills, then detected in SBT samples using molecular techniques [7,8,9]. Both species can act as serious pathogens by obstructing blood vessels and/or damaging gill epithelium in the circulatory system of SBT [10,11,12]. Untreated infections with these parasites can lead to mortalities [12]. The anthelmintic praziquantel (PZQ) was shown to be effective against *Cardicola* spp. and introduced by industry in 2012 [13]. Whilst PZQ treatment has reduced ranched SBT mortalities significantly, not all ranched SBT are treated, so ongoing monitoring of parasite loads can be a valuable tool to detect changes in infection severity as new treatment strategies are developed [14].

In 2018, a study was conducted on *Cardicola* spp. infection severity of SBT from a single commercial company from pontoons with different treatment strategies [14]. In 2019, an additional commercial company was sampled with a combination of treated and untreated pontoons. The aim of this paper is to investigate the effect of company, treatment, and ranching year on prevalence and intensity of *Cardicola* spp. infection in commercially ranched SBT.

## 2. Materials and Methods

### 2.1. Sample Collection and Processing

Wild SBT were purse seined in the Great Australian Bight (33°27′ S, 132°04′ E) and towed to the Spencer Gulf aquaculture zone near Port Lincoln, South Australia. SBT were sampled from two companies, Company A during the 2018 and 2019 ranching seasons, and Company B during the 2019 ranching season (Table 1). SBT samples collected during transfer from tow pontoon to ranching pontoon were referred to as week 0, and sampling time points thereafter reflect the number of weeks in ranching. Pontoons sampled from each company were from the same tow each year and stocked within 48 hours of arrival. All pontoons in this study were located in an area between 20–25 m water depth.

SBT from both companies were sampled from some PZQ treated and some untreated pontoons. For Company A in 2018, pontoon 1 was treated week 2 of ranching, pontoon 2 and 4 treated week 6 of ranching, and pontoon 3 was left untreated. For Company A in 2019, pontoon 1 was untreated, and pontoon 2 and pontoon 3 treated week 5 of ranching. Treated SBT from Company A were orally administered PZQ at a dose of 15–22 mg/kg bodyweight. For Company B in 2019, pontoons 1–3 were left untreated and pontoon 4 treated week 5 of ranching. Treated SBT from Company B were orally administered PZQ at a dose of 15–24 mg/kg bodyweight.

SBT were sampled using a baited hook and line from transfer (tow pontoon to ranching pontoon) to week 12. Divers caught SBT from week 17 to week 24. SBT were euthanized using the iki jime method. Fish were then gilled, gutted and wired, the entire process taking under a minute per fish. On shore, weight and total length for each SBT sampled were obtained. This allowed a condition index to be calculated using the South Australian tuna industry formula, whole weight (kg)/length (m)^3^. As SBT were sampled during commercial operations, whole weights were estimated from the following formula: gilled and gutted weight (kg)/0.87. SBT heart and gills were collected and processed using methods previously described [3,14]. Hearts were dissected 2–4 h after removal and flushed with water to dislodge adult flukes. Gill filaments were examined for eggs under a 40× compound microscope and quantified as eggs/mm filament length, taking an average from four filaments. Cumulative mortalities for each pontoon sampled were obtained (Table 2).

### 2.2. DNA Analysis

DNA was extracted from 25 mg of gill and heart samples preserved in RNAlater^®^ using the method and quality controls outlined in Power et al. 2019 [14]. The species-specific primers and probes targeting heterogeneous areas of the internal transcribed spacer-2 (ITS-2) region of rDNA used to detect *C. forsteri* and *C. orientalis* were designed in previous studies, which confirmed their specificity [9,15]. *Cardicola forsteri* and *C. orientalis* DNA was quantified using quantitative polymerase chain reaction (qPCR) techniques previously described [14].

### 2.3. Statistics

The effects of treatment, ranching year and company on severity of SBT infection with *C. forsteri* and *C. orientalis* were interpreted using GraphPad Prism 8 (GraphPad software, San Diego, CA, USA) as per Power et al. 2019 [14]. Briefly, severity of infection for *C. forsteri* and *C. orientalis* was described by prevalence and intensity as defined by Bush et al., where mean intensity is the average number of parasites per infected host [16]. Data did not meet the assumptions of normality so non-parametric tests were used.

The effect of treatment, ranching year, company and ranching time on infection prevalence was determined using Chi square analysis or Fisher’s exact test. Infection intensities were compared using an unpaired t-test or Kruskal–Wallis followed by Dunn’s or Mann–Whitney test for pairwise comparisons. The relationship between cumulative mortality and weeks in ranching was determined using simple linear regression. Chi square analysis was used to compare differences in cumulative mortality. Spearman’s rank correlation coefficients were used to determine the relationship between condition index and *Cardicola* spp. infection prevalence and intensity. Diagnostic method sensitivity was compared using a two-tailed McNemar χ^2^ test.

To determine the effects of treatment, samples from Company B in 2019 were used to directly compare treated and untreated pontoons at week 12 and week 17 of ranching. Samples from Company A in 2018 and 2019 were used to assess the effects of ranching year through direct comparison of untreated pontoons week 0 and week 3, and treated pontoons week 12 and week 17 of ranching. Samples from 2018 and 2019 were also compared over time. To determine the effects of company, samples from Company A in 2019 and Company B in 2019 were used to directly compare treated pontoons week 12 and week 17 of ranching. To compare untreated pontoons from Company A and Company B, samples were directly compared month 0, month 2 and month 5 of ranching. Pontoons from each company sampled within the same week/month were pooled if *p* > 0.25. Samples from Company A and Company B were also compared over time. Significance for all statistical analysis was assumed at *p* ≤ 0.05.

## 3. Results

### 3.1. Effect of Treatment

**Figure 1:**
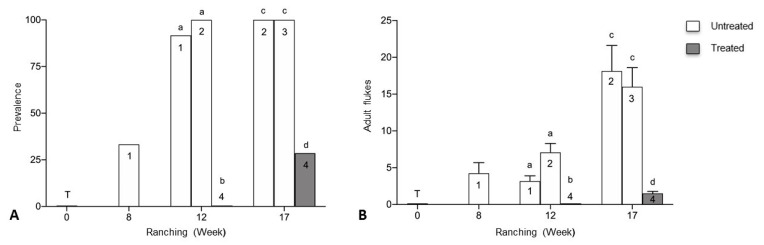
(**A**) Prevalence and (**B**) mean intensity (±SE) of adult *Cardicola forsteri* infection in heart from Company B during 2019 ranching of Southern Bluefin Tuna in Port Lincoln, South Australia (*n* = 12–15 for each pontoon at each time point). Pontoon 1, Pontoon 2 and Pontoon 3 were left untreated, Pontoon 4 treated with PZQ week 5. Numbers denote pontoon number. Different letters denote statistical differences at *p* ≤ 0.05 between pontoons at week 12 and week 17.

Prevalence and mean intensity of infection with adult *C. forsteri* was higher in untreated pontoons than treated in week 12 and week 17 of ranching (Figure 1). Prevalence increased for Pontoon 1 (untreated) from 33.3% week 8 to 91.7% week 12 and was significantly higher than Pontoon 4 (treated) week 12 with no adult *C. forsteri* detected (*p* < 0.0001). Prevalence for Pontoon 2 (untreated) was 100% week 12 and week 17, significantly higher than Pontoon 4 (treated) week 12 (*p* < 0.0001) and week 17 (*p* < 0.0001). Prevalence for Pontoon 3 (untreated) was 100% week 17, significantly higher than 28.6% prevalence for Pontoon 4 (treated) (*p* < 0.0001). Mean intensity of adult *C. forsteri* infection decreased for Pontoon 1 (untreated), from 4.25 (±1.44) week 8 to 3.18 (±0.72) week 12 and was significantly higher than Pontoon 4 (treated) week 12 with no adult *C. forsteri* detected (H = 26.66, *p* = 0.0024). Mean intensity for Pontoon 2 (untreated) increased from 7.08 (±1.22) week 12 to 18.13 (± 3.48) week 17 and was significantly higher than Pontoon 4 (treated) week 12 (H = 26.66, *p* < 0.0001) and week 17 (H = 10.38, *p* = 0.0054). Mean intensity for Pontoon 3 (untreated) was 16.00 (± 2.60) week 17, significantly higher than 1.50 (±0.29) for Pontoon 4 (treated) (H = 10.38, *p* =0 0.0094). No significant differences in prevalence or mean intensity were seen between untreated pontoons week 12 and week 17, and no adult *C. forsteri* were detected week 0 (transfer).

**Figure 2:**
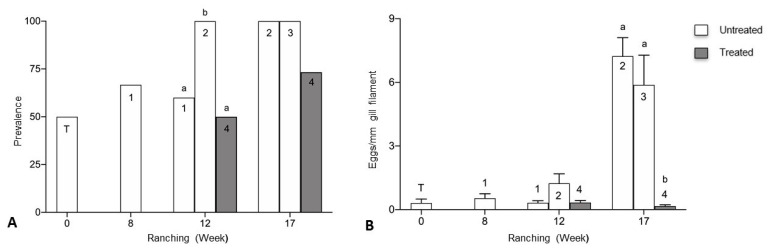
(**A**) Prevalence of *Cardicola* spp. eggs in gill filament; and (**B**) mean intensity (±SE) of *Cardicola* spp. egg/mm of gill filament from Company B during 2019 ranching of Southern Bluefin Tuna in Port Lincoln, South Australia (*n* = 12–15 for each pontoon at each time point). Pontoon 1, Pontoon 2 and Pontoon 3 were left untreated, Pontoon 4 treated with PZQ week 5. Numbers denote pontoon number. Different letters denote statistical differences at *p* ≤ 0.05 between pontoons at week 12 and week 17.

Prevalence of *Cardicola* spp. eggs in gill filaments was 50% at week 0 (transfer) (Figure 2A). Prevalence for Pontoon 1 (untreated) decreased from 66.7% week 8 to 60% in week 12. Prevalence for Pontoon 2 (untreated) was 100% in week 12 and week 17, significantly higher than Pontoon 1 (untreated) (*p* = 0.0287) and Pontoon 4 (treated) (*p* = 0.0137) in week 12. Prevalence for Pontoon 4 (treated) increased from 50% in week 12 to 73.3% in week 17. No significant differences in prevalence were seen between Pontoon 1 (untreated) and Pontoon 4 (treated) at week 12, or between Pontoons at week 17. Mean intensity of *Cardicola* spp. eggs/mm gill filament was 0.31 (± 0.19) at week 0 (transfer) (Figure 2B). Mean intensity for Pontoon 1 (untreated) decreased from 0.54 (±0.21) in week 8 to 0.32 (±0.10) in week 12. Mean intensity for Pontoon 2 increased from 1.24 (±0.45) week 12 to 7.24 (±0.87) week 17, significantly higher than Pontoon 4 (treated) (H = 25.18, *p* < 0.0001) week 17. Mean intensity for Pontoon 3 (untreated) was 5.88 (±1.40) in week 17, significantly higher than Pontoon 4 (treated) (H = 25.18, *p* = 0.0006) in week 17. Mean intensity for Pontoon 4 (treated) decreased from 0.33 (±0.10) in week 12 to 0.17 (±0.06) in week 17. No significant differences in mean intensity were seen between pontoons at week 12.

**Figure 3:**
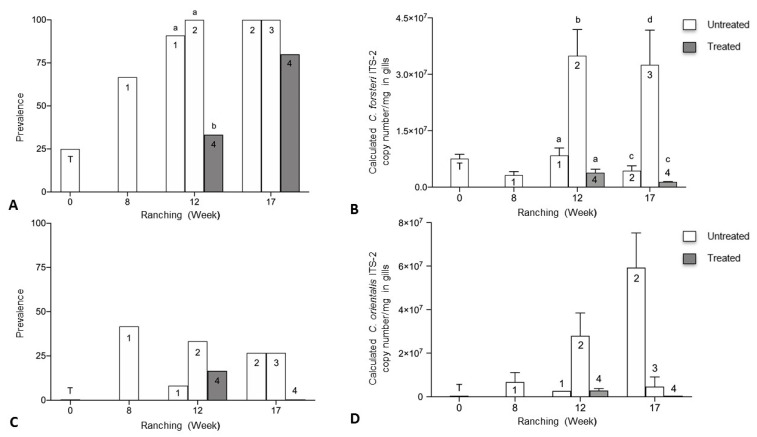
*Cardicola forsteri* (ITS-2) and *Cardicola orientalis* (ITS-2) in gills from Company B during 2019 ranching of Southern Bluefin Tuna in Port Lincoln, South Australia (*n* = 12–15 for each pontoon at each time point). (**A**) Prevalence of *Cardicola forsteri* (ITS-2) in gills; (**B**) mean calculated *Cardicola forsteri* (ITS-2) copy number/mg intensity (±SE) in gill samples; (**C**) prevalence of *Cardicola orientalis* (ITS-2) in gills; and (**D**) mean calculated *Cardicola orientalis* (ITS-2) copy number/mg intensity (±SE) in gill samples. Numbers denote pontoon number. Pontoon 1, Pontoon 2 and Pontoon 3 were left untreated, Pontoon 4 treated with PZQ week 5. Different letters denote statistical differences at *p* ≤ 0.05 between pontoons at week 12 and week 17.

Prevalence of *C. forsteri* (based on positive qPCR of ITS-2) in gill samples was significantly higher in untreated pontoons than treated at week 12 of ranching (Figure 3A). Prevalence increased for Pontoon 1 (untreated) from 66.7% in week 8 to 90.9% in week 12 and was significantly higher than Pontoon 4 (treated) in week 12 at 33.3% (*p* = 0.0094). Prevalence for Pontoon 2 (untreated) was 100% in week 12 and week 17, significantly higher than Pontoon 4 (treated) in week 12 (*p* = 0.0013). No differences were seen in prevalence between pontoons at week 17. Mean calculated *C. forsteri* (ITS-2) copy number/mg intensity in gill samples was significantly higher in Pontoon 2 (untreated) than Pontoon 1 (untreated) (H = 16.15, *p* = 0.0058) and Pontoon 4 (treated) in week 12 (H = 16.15, *p* = 0.0017) (Figure 3B). Mean intensity was significantly higher in Pontoon 3 (untreated) than Pontoon 2 (untreated) (H = 19.40, *p* = 0.0003) and Pontoon 4 (treated) in week 17 (H = 19.40, *p* = 0.0108). No differences were seen between Pontoon 1 (untreated) and Pontoon 4 (treated) in week 12, and Pontoon 2 (untreated) and Pontoon 4 (treated) in week 17.

*Cardicola orientalis* (ITS-2) was not detected in gill samples at transfer (Figure 3C). Prevalence decreased in Pontoon 1 (untreated) from 41.7% in week 8 to 8.3% in week 12, decreased in Pontoon 2 (untreated) from 33.3% in week 8 to 26.7% in week 12, and decreased in Pontoon 4 (treated) from 16.7% in week 12 to not being detected in week 17. Mean calculated *C. orientalis* (ITS-2) copy number/mg intensity in gill samples decreased in Pontoon 1 (untreated) from 6.81 × 10^6^ in week 8 to 2.70 × 10^6^ in week 12 and increased in Pontoon 2 (untreated) from 2.80 × 10^6^ in week 12 to 5.93 × 10^7^ in week 17 (Figure 3D). No differences were seen in prevalence and mean intensity of *C. orientalis* (ITS-2) in gill samples between pontoons at week 12 and week 17.

Mean condition index of ranched SBT was 23.5 (±1.32) at week 0 and no data were available at week 8. There was no difference between mean condition index of SBT from Pontoon 2 (untreated) (24.5 ± 0.92) and Pontoon 4 (treated) (23.1 ± 0.32) in week 12 (*p* = 0.1474). No data were available for Pontoon 1 (untreated) in week 12. There were no differences between mean condition index of SBT from Pontoon 2 (untreated) (24.5 ± 0.23), Pontoon 3 (untreated) (24.7 ± 0.67) and Pontoon 4 (treated) (25.0 ± 0.45) in week 17 (*p* = 0.5246).

### 3.2. Effect of Ranching Year

**Figure 4:**
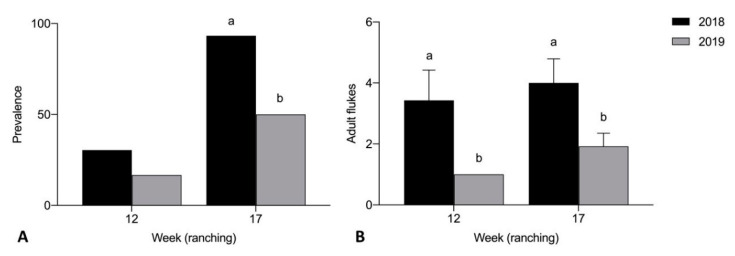
(**A**) Prevalence and (**B**) mean intensity (±SE) of adult *Cardicola forsteri* infection in heart from Company A treated pontoons during 2018 and 2019 ranching of Southern Bluefin Tuna in Port Lincoln, South Australia (week 12 2018 *n* = 24, week 12 2019 *n* = 12, week 17 2018 *n* = 15, week 17 2019 *n* = 27). Different letters denote statistical differences at *p* ≤ 0.05 between ranching years at week 12 and week 17.

Prevalence of adult *C. forsteri* in heart was significantly higher in 2018 than 2019 week 17 of ranching for Company A treated pontoons (*p* = 0.0061) (Figure 4A). Mean intensity of adult *C. forsteri* infection was significantly higher in 2018 than 2019 week 12 (U = 30, *p* = 0.0490) and week 17 (U = 51, *p* = 0.0416) of ranching for Company A treated pontoons (Figure 4B). No significant differences were seen in prevalence and mean intensity of *C. forsteri* infection between 2018 and 2019 Company A untreated pontoons week 0 and week 3. Prevalence of adult *C. forsteri* increased over time in 2018 treated pontoons (*p* = 0.0002) but no differences were seen in mean intensity over time in 2018, or in prevalence and mean intensity over time in 2019 treated pontoons.

**Figure 5:**
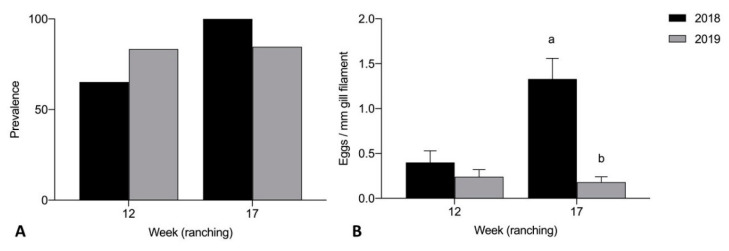
(**A**) Prevalence of *Cardicola* spp. eggs in gill filament; and (**B**) mean intensity (±SE) of *Cardicola* spp. egg/mm of gill filament from Company A treated pontoons during 2018 and 2019 ranching of Southern Bluefin Tuna in Port Lincoln, South Australia (week 12 2018 *n* = 24, week 12 2019 *n* = 12, week 17 2018 *n* = 15, week 17 2019 *n* = 27). Different letters denote statistical differences at *p* ≤ 0.05 between ranching years at week 12 and week 17.

Mean intensity of *Cardicola* spp. eggs in gill filament was significantly higher in 2018 than 2019 week 17 of ranching for Company A treated pontoons (U = 19, *p* < 0.0001) (Figure 5B). No significant differences were seen in prevalence of *Cardicola* spp. eggs in gills between Company A treated pontoons week 12 and week 17 (Figure 5A), mean intensity between Company A treated pontoons week 12, and prevalence and mean intensity between Company A untreated pontoons week 0 and week 3. Prevalence and mean intensity of *Cardicola* spp. eggs in gills increased over time in 2018 treated pontoons (*p* = 0.0148, U = 32, *p* = 0.0009) but no differences were seen in prevalence and mean intensity over time in 2019 treated pontoons.

**Figure 6:**
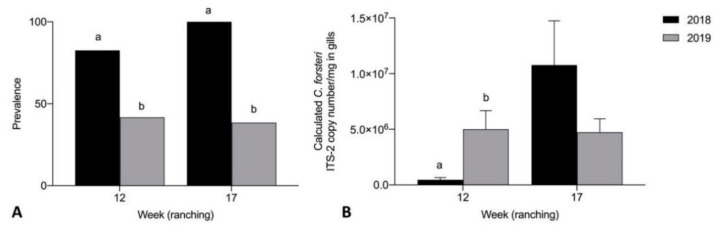
(**A**) Prevalence of *Cardicola forsteri* (ITS-2) in gills; and (**B**) mean calculated *Cardicola forsteri* (ITS-2) copy number/mg intensity (±SE) in gill samples from Company A treated pontoons during 2018 and 2019 ranching of Southern Bluefin Tuna in Port Lincoln, South Australia (week 12 2018 *n* = 24, week 12 2019 *n* = 12, week 17 2018 *n* = 15, week 17 2019 *n* = 27). Different letters denote statistical differences at *p* ≤ 0.05 between ranching years at week 12 and week 17.

Prevalence of *C. forsteri* (ITS-2) in gill samples was significantly higher in 2018 than 2019 week 12 (*p* = 0.0223) and week 17 (*p* = 0.0001) of ranching for Company A treated pontoons (Figure 6A). Mean calculated intensity of *C. forsteri* (ITS-2) copy number/mg in gill samples was significantly higher in 2019 than 2018 week 12 of ranching for Company A treated pontoons (U = 3, *p* = 0.0003) (Figure 6B). No significant differences were seen in prevalence and mean intensity between 2018 and 2019 Company A untreated pontoons week 0 and week 3. No significant differences were seen in prevalence and mean intensity of *C. orientalis* (ITS-2) in gills between 2018 and 2019 Company A untreated pontoons week 0 and week 3, and between 2018 and 2019 Company A treated pontoons week 12 and week 17. Mean calculated *C. forsteri* (ITS-2) copy number/mg intensity in gill samples increased over time in 2018 treated pontoons (U = 7, *p* < 0.0001). No differences were seen in prevalence over time in 2018 treated pontoons, or in prevalence and mean intensity over time in 2019 treated pontoons.

Mean condition index of ranched SBT sampled from untreated pontoons in 2018 was 17.6 (±0.19) at week 0, significantly higher than SBT sampled in 2019 with a mean condition index of 16.0 (±0.25) (U = 16, *p* = 0.0007). At week 3, mean condition index of SBT sampled from untreated pontoons in 2019 was 18.3 (±0.32), significantly higher than SBT sampled in 2018 with a mean condition index of 14.5 (±0.28) (U = 0, *p* < 0.0001). At week 12, mean condition index of SBT sampled in 2019 from treated pontoons was 24.1 (±0.41), significantly higher than SBT sampled in 2018 with a mean condition index of 22.3 (±0.33) (U = 53, *p* = 0.0023). At week 17, mean condition index of SBT sampled from treated pontoons in 2019 was 25.4 (±0.29), but no results for SBT condition index were obtained from treated pontoons in 2018.

### 3.3. Effect of Company

**Figure 7:**
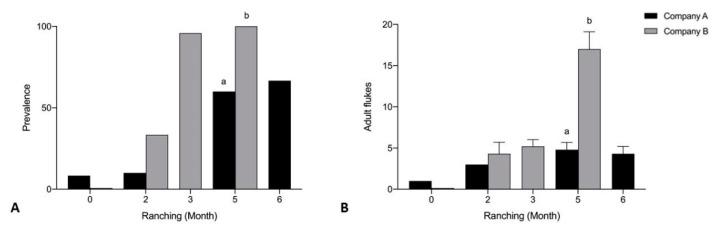
(**A**) Prevalence and (**B**) mean intensity (±SE) of adult *Cardicola forsteri* infection in heart from Company A and Company B untreated pontoons during 2019 ranching of Southern Bluefin Tuna in Port Lincoln, South Australia (A0 *n* = 12, B0 *n* = 12, A2 *n* = 10, B2 *n* = 12, A2 *n* = 14, B3 *n* = 24, A5 *n* = 15, B5 *n* = 30, A6 *n* = 15). Different letters denote statistical differences at *p* ≤ 0.05 between companies at each time point.

No significant differences were seen in prevalence and mean intensity of *Cardicola* spp. infection between Company A and Company B treated pontoons week 12 and week 17 of ranching for any diagnostic method. When comparing untreated pontoons from Company A and Company B, there were no time points (by week) to directly compare, so samples from each company were compared by ranching month. Prevalence and mean intensity of adult *C. forsteri* in 2019 was significantly higher for Company B than Company A untreated pontoons month 5 of ranching (*p* = 0.0006, U = 22, *p* < 0.0001) (Figure 7). No significant differences were seen in prevalence and mean intensity of *C. forsteri* infection between Company A and Company B untreated pontoons month 0 and month 2. Prevalence of adult *C. forsteri* increased over time for Company A (*p* = 0.0013) and Company B (*p* < 0.0001) untreated pontoons. Mean intensity of adult *C. forsteri* increased over time for Company B untreated pontoons (*p* < 0.0001), but no differences were seen for Company A.

**Figure 8:**
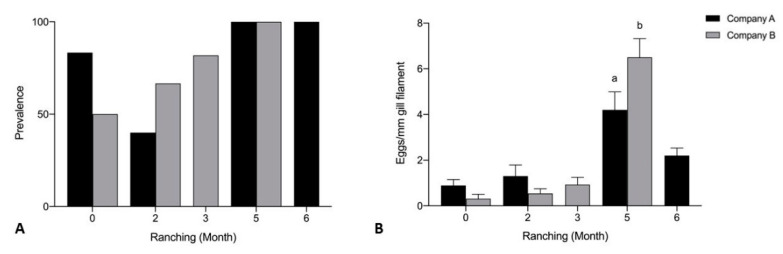
(**A**) Prevalence of *Cardicola* spp. eggs in gill filament; and (**B**) mean intensity (±SE) of *Cardicola* spp. egg/mm of gill filament from Company A and Company B untreated pontoons during 2019 ranching of Southern Bluefin Tuna in Port Lincoln, South Australia (A0 *n* = 12, B0 *n* = 12, A2 *n* = 10, B2 *n* = 12, A2 *n* = 14, B3 *n* = 24, A5 *n* = 15, B5 *n* = 30, A6 *n* = 15). Different letters denote statistical differences at *p* ≤ 0.05 between companies at each time point.

Mean intensity of *Cardicola* spp. eggs/mm gill filament was significantly higher for Company B than Company A untreated pontoons at month 5 of ranching (U = 143, *p* = 0.0489) (Figure 8). No significant differences were seen in mean intensity of *Cardicola* spp. eggs in gills between Company A and Company B untreated pontoons month 0 and month 2 of ranching, and prevalence of *Cardicola* spp. eggs in gills at any time point. Prevalence of *Cardicola* spp. eggs in gill filaments increased over time for Company A (*p* = 0.0158) and Company B (*p* = 0.0003) untreated pontoons. Mean intensity of *Cardicola* spp. eggs/mm gill filament increased over time for Company B untreated pontoons (*p* < 0.0001), but no differences were seen for Company A.

**Figure 9:**
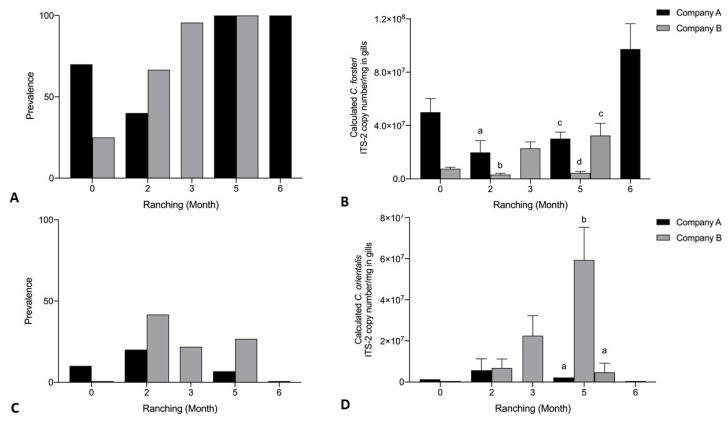
*Cardicola forsteri* (ITS-2) and *Cardicola orientalis* (ITS-2) in gills from Company A and Company B untreated pontoons during 2019 ranching of Southern Bluefin Tuna in Port Lincoln, South Australia (A0 *n* = 12, B0 *n* = 12, A2 *n* = 10, B2 *n* = 12, A2 *n* = 14, B3 *n* = 24, A5 *n* = 15, B5 *n* = 30, A6 *n* = 15). (**A**) Prevalence of *Cardicola forsteri* (ITS-2) in gills; (**B**) mean calculated *Cardicola forsteri* (ITS-2) copy number/mg intensity (±SE) in gill samples; (**C**) prevalence of *Cardicola orientalis* (ITS-2) in gills; and (**D**) mean calculated *Cardicola orientalis* (ITS-2) copy number/mg intensity (±SE) in gill samples. Different letters denote statistical differences at *p* ≤ 0.05 between companies at each time point.

Mean calculated *C. forsteri* (ITS-2) copy number/mg in gill samples was significantly higher for Company A than Company B untreated pontoons month 2 of ranching (U = 2, *p* = 0.0162), and significantly higher for Company A than a single Company B untreated pontoon month 5 of ranching (H = 22.47, *p* < 0.0001) (Figure 9B). Company B untreated pontoons could not be pooled at month 5 as one pontoon was significantly higher than the other (H = 22.47, *p* = 0.0003). Mean calculated *C. orientalis* (ITS-2) copy number/mg in gill samples was significantly higher for a single Company B pontoon than Company A untreated pontoons month 5 of ranching (H = 5.07, *p* < 0.0001) (Figure 9D). Company B untreated pontoons could not be pooled at month 5 as one pontoon was significantly higher than the other (H = 3.70, *p* < 0.0001). No significant differences were seen in prevalence of *C. forsteri* (ITS-2) or *C. orientalis* (ITS-2) in gill samples between Company A and Company B untreated pontoons (Figure 9A,C). Prevalence of *C. forsteri* (ITS-2) copy number/mg in gill samples increased over time for Company A (*p* = 0.0002) and Company B (*p* < 0.0001) untreated pontoons. Mean intensity of *C. forsteri* (ITS-2) copy number/mg in gill samples increased over time for Company A (H = 15.89, *p* = 0.0022) and Company B (H = 30.32, *p* = 0.0043) untreated pontoons. Prevalence and intensity of *C. orientalis* (ITS-2) copy number/mg in gill samples did not increase over time for Company A or Company B.

There were no significant differences in mean condition index of ranched SBT between Company A and Company B treated pontoons. Mean condition index of ranched SBT sampled from treated pontoons was 24.1 (±0.41) for Company A and 23.1 (±0.32) for Company B week 12. Mean condition index of ranched SBT sampled from treated pontoons was 25.4 (±0.29) for Company A and 25.0 (±0.45) for Company B week 17. When comparing untreated pontoons, mean condition index of ranched SBT sampled from Company B (23.5 ± 1.31) was significantly higher than Company A (17.5 ± 0.25) at month 0 (*p* < 0.0001). No differences were seen between mean condition of ranched SBT from Company A (23.7 ± 0.38) and Company B (24.6 ± 0.35) untreated pontoons month 5 of ranching. Mean condition index could not be compared month 2 of ranching as some results were not available.

### 3.4. Cumulative Mortality

There was no relationship between cumulative mortality and weeks in ranching for SBT pontoons sampled in this study (d.f. = 9, R^2^ = 0.002, *p* = 0.8738). Cumulative mortality for Company B, Pontoon 2 (untreated) in 2019 was significantly higher than all other pontoons sampled (Chi-square X^2^ = 40.16, d.f. = 9, *p* < 0.0001). No differences in cumulative mortality were seen between treated and untreated pontoons in 2019 for Company A, or between 2018 and 2019 pontoons for Company A.

### 3.5. Effect of Cardicola *spp.* Infection Severity on SBT Condition Index

There was a significant positive correlation between SBT condition index and adult *C. forsteri* numbers in the heart for Company A (Spearman’s r = 0.6194, *p* < 0.001) and Company B (Spearman’s r = 0.2961, *p* = 0.008). There was a significant positive correlation between SBT condition index and *Cardicola* spp. eggs/mm gill filament for Company A (Spearman’s r = 0.2553, *p* = 0.007) and Company B (Spearman’s r = 0.2605, *p* = 0.019). A significant positive correlation was also seen between SBT condition index and *C. forsteri* ITS-2 copy number/mg for Company A (Spearman’s r = 0.3718, *p* < 0.001). No correlations were found between SBT condition index and *C. forsteri* ITS-2 copy number/mg for Company B or between SBT condition index and *C. orientalis* ITS-2 copy number/mg for both companies.

### 3.6. Comparison between Cardicola *spp.* Diagnostic Methods

For *C. forsteri* in SBT hearts, detection of *C. forsteri* using ITS-2 qPCR had a higher sensitivity than detection of *C. forsteri* using heart flush microscopy (McNemar’s test χ2 test, n = 238, *p* < 0.0001) (Table 3). For the presence of *Cardicola* spp. in SBT gills, detection of *C. forsteri* and *C. orientalis* using ITS-2 qPCR showed similar sensitivity to detection of *Cardicola* spp. eggs using gill microscopy (McNemar’s test χ2 test, n = 308, *p* = 0.4185) (Table 4).

## 4. Discussion

Results from this study indicate PZQ remains highly effective against *C. forsteri* infection. When comparing treated and untreated pontoons (Company B) in 2019, prevalence of *C. forsteri* infection in SBT was significantly higher in untreated pontoons week 12 of ranching (7 weeks post treatment), and mean intensity significantly higher in untreated pontoons week 12 and week 17 (12 weeks post treatment). It is likely PZQ is also effective against *C. orientalis* infection, however prevalence was too low to determine its efficacy based on epidemiological data, and the pharmacokinetics of PZQ on adult *C. orientalis* has not been studied previously [13,17]. Results from epidemiological studies help industry to develop the best management strategies for diseases, including treatment, with some companies reducing the number of pontoons treated, thereby reducing their costs. Keeping some pontoons untreated may maintain a population of blood flukes that remain susceptible to anthelmintics in future years [18,19]. Continuing to monitor blood fluke infections in ranched SBT will help to detect changes in drug efficacy over time, and guide future treatment strategies for industry.

When investigating the effect of ranching year, lower prevalence and intensity of *Cardicola* spp. infection was seen in 2019 than 2018 for Company A treated pontoons week 12 and week 17 of ranching. However, when comparing untreated pontoons from earlier in the season (week 0 and week 4), there were no significant differences. Prevalence and intensity of *Cardicola* spp. infection increased from week 12 to week 17 in 2018 treated pontoons, but there were no differences from week 12 to week 17 in 2019. These results may be an indication that consistent years of PZQ treatment have lowered the parasite load in the environment, making re-infection after treatment less likely. Given this study compares only two years of data and other environmental or health factors may be involved, sampling over 3+ years could determine if there are any long-term trends over ranching years in prevalence and intensity of blood fluke infections in ranched SBT or if these results are simply due to annual variation.

There were no differences between treated pontoons for Company A and Company B. Given both companies use similar treatment strategies, their lease sites are located at similar water depths, and there were no differences in SBT condition, these results are not surprising. When comparing untreated pontoons, prevalence and mean intensity of adult *C. forsteri*, and mean intensity of *Cardicola* spp. eggs was significantly higher for Company B month 5 of ranching. Mean intensity of *C. forsteri* (ITS-2) DNA varied between Company B pontoons at month 5 of ranching, and only one pontoon was significantly higher than Company A. It is interesting to note that mean intensity of adult *C. forsteri* and *Cardicola* spp. eggs increased over time for Company B but not for Company A. These results may be further indication that re-infection is less likely in the environment near Company A pontoons. In a long-term survey of ranched SBT sampled over three ranching seasons (2004–2006), the universal factor explaining *C. forsteri* infection prevalence, abundance and intensity was Company [4]. More specifically, differences between companies may be related to differences in husbandry methods, different sizes of SBT caught for ranching, or the location of lease sites. Our study was completed during commercial operations so was limited with the number of companies and time points included. Given there are now differences in PZQ treatment strategies between companies to consider, another long-term study sampling several companies is warranted.

No differences in cumulative mortality were seen between treated and untreated pontoons, or between ranching years for Company A. However, cumulative mortality for Company B pontoon 2 (untreated) in 2019 was significantly higher than all other pontoons sampled at 1.82%. As the cause of mortality is unknown, it is difficult to determine the reason for this difference. Company B pontoon 2 also had the highest mean intensity of *C. orientalis* ITS-2 DNA in gill samples in this study. In Pacific Bluefin Tuna *Thunnus orientalis*, *C. orientalis* has been shown to have higher pathogenicity than *C. opisthorchis* and *C. forsteri* as it produces significantly more eggs in the gills, and the adults clog the branchial arteries and restrict blood flow [11]. When mortalities peaked in ranched SBT, the dominant species detected was *C. orientalis* [6,9]. It is possible that a higher intensity of *C. orientalis* could contribute to the higher mortality rate seen in Company B pontoon 2.

SBT condition index varied between ranching year and company at certain time points, however no consistent differences were seen. For example, SBT condition index was higher for Company A in 2018 at week 0, but higher in 2019 at week 3 and week 12. This variation may be due to low sample size. There are limitations when studying the relationship between *Cardicola* spp. infection and condition of SBT, as the same fish can only be sampled once throughout the ranching season. Results indicate the severity of infection seen in this study was too low to have an effect on condition of fish.

Molecular diagnostics are crucial when studying infection dynamics of *Cardicola* spp. in ranched SBT. Mean number of *Cardicola* spp. eggs in gills was relatively low in Pontoon 2 (untreated) week 12, but mean intensity of *C. forsteri* (ITS-2) and *C. orientalis* (ITS-2) in gill samples from the same fish was higher in comparison. Egg counts may be low due to the life cycle stage when sampling (miracidia may have hatched from eggs), or due to an uneven distribution of eggs in gill filaments sampled [11,20]. Mean intensity of *Cardicola* spp. eggs in gills was relatively high in Pontoon 2 (untreated) week 17 and in comparison, mean intensity of *C. forsteri* (ITS-2) was relatively low in gill samples but mean intensity of *C. orientalis* (ITS-2) was relatively high. When comparing diagnostic methods, qPCR had higher sensitivity for detection of *C. forsteri* in SBT heart than microscopy. Sensitivity was similar when comparing diagnostic methods for detection of *Cardicola* spp. in gills, but qPCR offers more specificity with *Cardicola* species differentiation [9].

Traditional methods, such as adult counts of *C. forsteri* from SBT heart and egg counts from SBT gills, are simple to perform and can be field based [3,21]. However, traditional methods are laborious, and only detect one life stage—either adults or eggs [10,11]. Additionally, detection of adult *C. orientalis* is difficult using traditional methods [22]. The use of qPCR for detection of *Cardicola* spp. shows greater sensitivity and specificity than traditional methods, but it requires specialised training and equipment, and access to a laboratory [14,16]. These disadvantages negate the potential time advantage afforded by molecular diagnostics. Future research should develop a field-based method that combines the precision of molecular methods with the ease of traditional methods, e.g., use of isothermal amplification assays [23]. Isothermal amplification assays such as recombinase polymerase amplification (RPA), demonstrate optimal temperature at 37 °C and can be coupled with a lateral flow strip, making it suitable for rapid diagnosis onshore with limited equipment [23].

Studying infection dynamics of *Cardicola* spp. in ranched SBT is important given there may be differences in pathogenesis between species [11,14]. Since 2013, *C. forsteri* has been the dominant species documented in ranched SBT populations [14,16]. From 2008 to 2012, *C. orientalis* was identified as the main species in SBT [9]. This change in species dynamic also corresponded with the introduction of PZQ treatment by industry, and a lower rate of mortalities seen during the ranching season [14,16]. Results from this study show *C. forsteri* continues to be the dominant species in ranched SBT, and overall prevalence of *C. orientalis* remains low. The reason for the decline of *C. orientalis* in SBT is unclear, as we do not fully understand the pharmacokinetics of PZQ on *C. orientalis*, and the lifecycle of this species as the intermediate host for *C. orientalis* has not yet been discovered in Australia.

Removal of the intermediate host is another option to control blood fluke infections in ranched SBT [24]. However, there are gaps in our knowledge around life cycles of *Cardicola*. In Australia, the asexual stages of *C. forsteri* were found in a single terebellid polychaete, *Longicarpus modestus*, near SBT pontoon sites [25]. In Japan, life cycles have been elucidated for *C. orientalis*, *C. forsteri* and *C. opisthorchis* with terebellid polychaetes identified as the intermediate host [26,27,28]. Research has also looked to determine spatial and temporal changes of terebellid polychaetes near tuna pontoons, as this can have different implications for potential efforts to control the parasite [29,30]. Information about the development of *Cardicola* spp. within intermediate hosts is also very limited. Recent work involving transplantation trials of *C. opisthorchis* and *C. orientalis* has opened the possibility for cultivation of *Cardicola* spp. in the laboratory [31,32]. Future work should look to elucidate the life cycle of *C. orientalis* in Australia; in particular, to identify the intermediate host, and determine what factors influence spatial and temporal changes of the intermediate host/s.

## Figures and Tables

**Table 1:** Sampling information including pontoon characteristics and Southern Bluefin Tuna collected from Company A in 2018 and 2019 and Company B in 2019. NA—not applicable.

	Transfer Date	Treatment Date	Treatment Dose (mg/kg)	Sampling Size						
				Week 0	Week 3	Week 8	Week 12	Week 17	Week 21	Week 24
COMPANY A 2018										
Transfer				12	-	-	-	-	-	-
Pontoon 1	28 Feb	9 Mar (Week 2)	15	-	12	12	12	-	-	-
Pontoon 2	28 Feb	3 Apr (Week 6)	15	-	12	12	12	-	-	-
Pontoon 3	28 Feb	NA	NA	-	12	12	12	-	-	-
Pontoon 4	28 Feb	3 Apr (Week 6)	15	-	-	-	-	15	-	-
COMPANY A 2019										
Transfer				12	-	-	-	-	-	-
Pontoon 1	8 Mar	NA	NA	-	10	-	-	-	15	15
Pontoon 2	9 Mar	9 Apr (Week 5)	15-22	-	-	-	12	12	-	-
Pontoon 3	7 Mar	11 Apr (Week 5)	15-22	-	-	-	-	15	-	-
COMPANY B 2019										
Transfer				12	-	-	-	-	-	-
Pontoon 1	28 Mar	NA	NA	-	-	12	12	-	-	-
Pontoon 2	28 Mar	NA	NA	-	-	-	12	15	-	-
Pontoon 3	26 Mar	NA	NA	-	-	-	-	15	-	-
Pontoon 4	27 Mar	29 Apr (Week 5)	15-24	-	-	-	12	15	-	-

**Table 2:** Cumulative mortalities from transfer to harvest of ranched Southern Bluefin Tuna in sampled pontoons from Company A in 2018 and 2019 and Company B in 2019.

	Cumulative Mortality (%)	Weeks in Ranching	Total Stocked Fish
COMPANY A 2018			
Pontoon 1	0.16	16	3128
Pontoon 2	0.31	16	3205
Pontoon 3	0.20	12	2545
Pontoon 4	0.43	17	3108
COMPANY A 2019			
Pontoon 1	0.10	24	2003
Pontoon 2	0.43	17	2082
Pontoon 3	0.43	17	3239
COMPANY B 2019			
Pontoon 1	0.46	15	1525
Pontoon 2	1.82	17	2808
Pontoon 3	0.54	17	2771
Pontoon 4	0.49	17	2849

**Table 3:** Comparison of diagnostic methods for *C. forsteri* in heart of the same individual fish (McNemar’s test χ^2^, *p* < 0.0001).

	*Cardicola forsteri* (ITS-2) in Heart Samples			
COMPANY A 2018				
Adult *Cardicola forsteri* in heart		+	−	Total
	+	78	7	85
	−	94	59	153
	Total	172	66	238

**Table 4:** Comparison of diagnostic methods for *Cardicola* spp. in gills of the same individual fish (McNemar’s test χ^2^, *p* < 0.4185).

	*Cardicola* spp. (ITS-2) in Gill Samples			
COMPANY A 2018				
*Cardicola* spp. eggs in gill filament		+	−	Total
	+	218	24	242
	−	31	35	66
	Total	249	59	308
